# Dr. Oliver Wendell Holmes

**Published:** 1894-11

**Authors:** 


### DR. OLIVER WENDELL HOLMES.

Dr. O. W. Holmes, of Boston, physician, author, and poet, died October 7th. He was a distinguished representative of both American medicine and American literature, and was an honor to both professions. His attention to these professions for many years was almost equally divided. He graduated at the Harvard Medical School in 1836, and in the same year issued his first book of poems.

In the next year he was awarded three of the four Boylston prizes (Harvard) offered for medical papers. In 1842, he delivered some "Lectures on Homeopathy and its Kindred Delusions," which are still classical. In 1847 Dr. Holmes was called to the chair of Anatomy at Harvard, but inasmuch as this "chair" included also physiology and microscopy, he used to speak of it as a "whole settee." The most important medical paper of his early career (1843) was entitled, "Puerperal Fever as a Private Pestilence," in which he announced the infectious character of the disease many years in advance of other writers. After 1855 his work was mainly in the field of literature, except that he continued his professorship of anatomy at Harvard. He was a thorough anatomist and an excellent teacher. If his lectures on anatomy betrayed the man of letters, so do his general writings give evidence of his medical training. In his "Elsie Venner" and "Mortal Antipathy," which he himself called "medicated novels," there are discussions of certain interesting medical questions enveloped in charming fiction.

Dr. Holmes's greatest work was the "Autocrat of the Breakfast Table," which he began in 1857 in the *Atlantic Monthly*. This book possesses an unique place in English literature. It cannot be described, but it contains many of his happiest thoughts and prettiest poems. Among his best poems are the "Last Leaf," the "Chambered Nautilus," and "The Voiceless."

Two months ago Dr. Holmes passed his eighty-fifth birthday, but he never knew age. He died like he had lived, a philosopher, an optimist, happy and contented, and beloved of everybody. He survived nearly all his early associates in Harvard University, and of the three, Bismarck, Tennyson, and himself, all the same age, he held out next to the last. The following selection from his own works is particularly applicable to himself:

As hour by hour his lengthened days declined,
A sweeter radiance lingered o'er his mind;
Cold were the lips that spoke his early praise,
And hushed the voices of his morning days,
Yet the same accents dwelt on every tongue,
And love renewing kept him ever young.

